# Recruiting hard-to-reach populations for surveys: A case of people with schizophrenia and coexisting diabetes

**DOI:** 10.1192/j.eurpsy.2025.459

**Published:** 2025-08-26

**Authors:** S. T. Rønne, B. Cleal, P. H. Gæde, S. M. Arnfred, R. Jørgensen

**Affiliations:** 1 Research Unit of Psychiatry West, Region Zealand, Slagelse; 2 Department of Clinical Medicine, Faculty of Health and Medical Sciences, University of Copenhagen; 3 Steno Diabetes Center Copenhagen, Copenhagen University Hospital, Copenhagen; 4 Department of Cardiology and Endocrinology, Slagelse Hospital, Slagelse; 5 Department of Regional Health Research, University of Southern Denmark, Odense; 6 Psychiatric Research Unit, Region Zealand, Slagelse; 7 Unit for Psychiatric Research, Aalborg University Hospital; 8 Department of Clinical Medicine, Aalborg University, Aalborg, Denmark

## Abstract

**Introduction:**

In research, recruitment challenges are common and lead to delays and reduce sample size and power. People with schizophrenia are often described as hard to reach and retain in research, and in particular, studies targeting people with chronic comorbidities such as diabetes, meet difficulties related to recruitment.

**Objectives:**

This study aims to describe challenges and strategies to recruitment of Danish adults with schizophrenia and type 2 diabetes to a cross-sectional survey study about psychosocial health and support.

**Methods:**

The recruitment process was tracked in a register where all relevant information was synthesized systematically. This included information on how eligible participants were identified and invited for the study and reasons for declining. Two recruitment strategies were applied for recruiting participants to complete a questionnaire: 1) Through mental health professionals in psychiatric outpatient clinics in Region Zealand, Denmark, and 2) Through phone calls to eligible participants. Descriptive analyses of the recruitment data were conducted.

**Results:**

Three types of challenges were found and described: 1) Identifying eligible participants, 2) Challenges with having mental health professionals to recruit, and 3) Participants’ lack of ability to complete a questionnaire. The challenges were met by several practical approaches: 1) Identifying eligible participants though electronic health records and medication types, 2) Inviting participants through phone calls, and 3) Letting participants receiving help for completing the questionnaire from a care coordinator, family/friend or researcher when needed. Approximately 15% of all eligible participants declined to take part, which indicate high willingness to participate.

**Conclusions:**

Exploring different types of challenges was important for understanding the actual difficulties in recruitment, for using approaches to meet the challenges, and for detecting the high willingness to take part.

**Disclosure of Interest:**

None Declared

